# 减低强度预处理单份非血缘脐血干细胞移植治疗范可尼贫血3例并文献复习

**DOI:** 10.3760/cma.j.issn.0253-2727.2022.08.012

**Published:** 2022-08

**Authors:** 丽娜 刘, 闿迪 宋, 宝林 汤, 湘 皖, 良权 耿, 雯 姚, 光宇 孙, 会兰 刘, 小玉 朱, 自敏 孙

**Affiliations:** 中国科学技术大学附属第一医院（安徽省立医院）血液科，合肥 230001 Department of Hematology, the First Affiliated Hospital of University of Science and Technology of China (Anhui Provincial Hospital), Hefei 230001, China

范可尼贫血（Fanconi anemia, FA）是一种罕见的常染色体隐性遗传性血液病。1988年，Gluckman等[1]在法国巴黎圣路易医院采用HLA相合同胞脐血干细胞移植治疗1例5岁的FA患儿并获得成功，从此拉开了脐血干细胞移植治疗白血病、骨髓衰竭性疾病以及一些先天性疾病的序幕。国内采用非血缘脐血干细胞移植治疗FA未见报道。本中心采用减低强度预处理单份非血缘脐血干细胞移植成功治疗3例FA患者，报告如下。

## 病例资料

例1，男，12岁，自幼全血细胞减少，2012年（6岁）诊断FA，诊断时HGB 89 g/L，PLT 24×10^9^/L，FANCD2基因杂合突变、ITGB2基因突变和MPL基因突变阳性。2018年5月就诊于我院，因缺乏同胞相合供者，拟行脐血干细胞移植。预处理方案：氟达拉滨（Flu）40 mg·m^−2^·d^−1^、−6 d～−2 d，环磷酰胺（Cy）10 mg·kg^−1^·d^−1^、−5 d～−2 d，全身照射（TBI）3 Gy、−7 d。预防移植物抗宿主病（GVHD）方案：环孢素A（CsA）3 mg·kg^−1^·d^−1^静脉点滴、−1 d开始，霉酚酸酯（MMF）25 mg·kg^−1^·d^−1^分次口服、+1 d开始。2018年9月7日回输脐血干细胞（高分辨HLA配型5/6、7/8、9/10、9/12位点相合），总有核细胞数（TNC）为1.84×10^7^/kg，CD34^+^细胞1.3×10^5^/kg。+12 d中性粒细胞植入，+25 d血小板植入。+14 d供者细胞嵌合率为99.7％，出现Ⅰ度急性GVHD（皮疹），加用甲泼尼龙1 mg·kg^−1^·d^−1^后好转；+32 d巨细胞病毒（CMV）DNA 3.49×10^2^/拷贝数；+33 d发生出血性膀胱炎，加用碳酸氢钠、利尿剂及免疫抑制剂减量后好转。移植后1年停用免疫抑制剂，移植后3年血常规、骨髓正常、致病基因阴性，已上中学。

例2，女，10岁。自幼“全血细胞减少”，2015年（7岁）诊断FA，诊断时HGB 58 g/L，PLT 14×10^9^/L，FANCA基因杂合突变，外周血染色体核型分析示16号染色体断裂异常，输血依赖。2018年7月就诊我院，无同胞相合供者，拟行脐血干细胞移植，预处理及预防GVHD方案同例1。2018年10月31日回输脐血（HLA 5/6、6/8、8/10、10/12位点相合），TNC 7.06×10^7^/kg，CD34^+^细胞4.02×10^5^/kg）。+21 d粒细胞植入，+30 d血小板植入。+14 d供者细胞嵌合率为96.9％；+17 d发热，面颈部出现皮疹，加用甲泼尼龙1 mg·kg^−1^·d^−1^治疗，皮疹控制不佳并出现腹泻，加用巴利昔单抗及甲氨蝶呤后症状好转；+27 d查CMV-DNA 1.01×10^2^/拷贝数，+43 d查CMV-DNA 1.0×10^4^/拷贝数，加用更昔洛韦等抗病毒治疗后转阴。移植后6个月停用免疫抑制剂，移植后7个月发生慢性GVHD（口腔溃疡），加用甲泼尼龙12 mg/d，症状控制后减停。随访至移植后3年，血常规、骨髓象及染色体核型均正常，已上学。

例3，女，4岁。自幼“左手六指伴全血细胞减少”，2019年（2岁）诊断FA，FANCE基因错义突变阳性，输血依赖。无同胞相合供者，拟行脐血干细胞移植。预处理及预防GVHD方案同例1，2020年5月回输脐血（HLA 6/6、8/8、10/10、10/12位点相合），TNC 7.5×10^7^/kg，CD34^+^细胞5.6×10^5^/kg。+15 d粒细胞植入，+14 d血小板植入。+3 d发热，+6 d出现皮疹，给予甲泼尼龙治疗后症状缓解；+11 d CMV-DNA 1.25×10^2^/拷贝数，+22 d CMV-DNA 3.16×10^2^/拷贝数；+14 d供者细胞嵌合率为100％。移植后6个月停用免疫抑制剂，移植后1年随访血常规、骨髓正常，致病基因转阴，无慢性GVHD，患儿已上幼儿园。

## 讨论及文献复习

1988年世界上首例采用同胞脐血干细胞移植治疗1例5岁FA患儿获得成功，+22 d粒细胞植入，发生Ⅰ度急性GVHD（皮肤），+47 d好转，无慢性GVHD，患者此后生长发育正常，成年后结婚生子[1]。

异基因造血干细胞移植（allo-HSCT）是根治FA的最好方法，但是大多数FA患者缺乏HLA相合同胞供者，因此替代供者移植是近几十年来学者研究的热点。无关供者骨髓移植（BMT）曾是FA患者的治疗选择[2]，但是GVHD发生率和治疗相关死亡率较高，总生存（OS）率仅为30％，25％～30％的患者出现植入失败（GF），50％～70％的患者出现预处理不良反应、GVHD和感染等并发症[3]。随着移植技术的改进，2015年明尼苏达大学报道2006至2012期间采用TBI+Flu+Cy+ATG预处理方案治疗48例FA患者（BMT 32例、脐血干细胞移植16例），移植后3年OS率为94％[3]。Gluckman等[4]回顾性分析了93例行脐血干细胞移植的FA患者，中位移植年龄为8.6（1～45）岁，得出预处理方案中加入Flu可使粒细胞植入率明显提高的结论。

提高FA患者移植疗效需要解决二个问题，一是提高植入率，二是降低移植相关死亡率。对于FA患者发病时多伴有多脏器损伤，输血依赖导致铁过载进一步加重脏器损伤和感染的发生。本研究的3例患儿移植前均有输血依赖。我们使用HLA高分辨配型≥5/6个位点相合脐血作为干细胞来源并采用减低强度预处理方案，GVHD预防方案未使用抗胸腺细胞球蛋白（ATG）以保护脐血中有限的T淋巴细胞，3例患儿均获得稳定造血重建，移植后无严重并发症发生，如正常儿童一样上学及生活，提示单份脐血干细胞移植可作为无同胞相合供者FA患儿的替代选择。
